# Disposition and effect of intra-articularly administered dexamethasone on lipopolysaccharide induced equine synovitis

**DOI:** 10.1186/s13028-019-0464-2

**Published:** 2019-06-20

**Authors:** Carl Ekstrand, Ulf Bondesson, Ellen Giving, Mikael Hedeland, Carina Ingvast-Larsson, Stine Jacobsen, Maria Löfgren, Lars Moen, Marie Rhodin, Tonje Saetra, Birgit Ranheim

**Affiliations:** 1 Department of Biomedical Sciences and Veterinary Public Health, Faculty of Veterinary Medicine and Animal Science, Swedish University of Agricultural Sciences, Box 7058, 750 07 Uppsala, Sweden; 2 Department of Chemistry, Environment and Feed Hygiene, National Veterinary Institute (SVA), 751 89 Uppsala, Sweden; 3 Department of Medicinal Chemistry, Faculty of Pharmacy, Uppsala University, Box 574, 751 23 Uppsala, Sweden; 4 Romerike Hesteklinikk, Riisveien 75, 2007 Kjeller, Norway; 5 Department of Veterinary Clinical Sciences, Faculty of Health and Medical Sciences, University of Copenhagen, Dyrlægevej 16, 1870 Copenhagen, Frederiksberg C Denmark; 6 Department of Anatomy, Physiology and Biochemistry, Faculty of Veterinary Medicine and Animal Science, Swedish University of Agricultural Sciences, Box 7011, 750 07 Uppsala, Sweden; 7 Rikstotoklinikken Bjerke AS., Postboks 194, 0510 Oslo, Norway; 8 Production Animal Clinical Science, Faculty for Veterinary Medicine, The Norwegian University of Life Sciences, Box 369 sentrum, 0102 Oslo, Norway

**Keywords:** Corticosteroids, Pharmacokinetics, Pharmacodynamics, Quantitative pharmacology

## Abstract

**Background:**

Dexamethasone is used for the intra-articular route of administration in management of aseptic arthritis in horses. Despite its widespread use there is very little quantitative data of the disposition and response to dexamethasone. The aim of this study was to investigate and describe the synovial fluid and plasma dexamethasone concentration over time and to explore the relation between synovial fluid concentration and response using clinical endpoints as response biomarkers after IA injection of dexamethasone disodium salt solution in an equine model of synovitis.

**Results:**

Inflammation was induced in the radiocarpal joint of six horses by injection of 2 ng lipopolysaccharide (LPS). Two hours later either saline or dexamethasone was injected in the same joint in a two treatment cross over design. Each horse was treated once with one of the six doses dexamethasone used (0.01, 0.03, 0.1, 0.3, 1 or 3 mg) and once with saline. Dexamethasone was quantified by means of UHPLC–MS/MS. Dexamethasone disposition was characterised by means of a non-linear mixed effects model. Lameness was evaluated both objectively with an inertial sensor based system and subjectively scored using a numerical scale (0–5). Joint circumference, skin temperature over the joint and rectal temperature were also recorded. The LPS-challenge induced lameness in all horses with high inter-individual variability. Dexamethasone significantly decreased lameness compared with saline. Other variables were not statistically significant different between treatments. Objective lameness scoring was the most sensitive method used in this study to evaluate the lameness response. A pharmacokinetic/pharmacodynamic model was successfully fitted to experimental dexamethasone and lameness data. The model allowed characterization of the dexamethasone synovial fluid concentration–time course, the systemic exposure to dexamethasone after intra-articular administration and the concentration–response relation in an experimental model of synovitis.

**Conclusions:**

The quantitative data improve the understanding of the pharmacology of dexamethasone and might serve as input for future experiments and possibly contribute to maintain integrity of equine sports.

## Background

Joint injury or disease is common in both equine athletes and companion horses. In racehorses, the overall injury rate was estimated to 1.8 injuries per 100 months the horses were at risk for injuries, i.e. from entering the study until diagnosed with an injury [1]. Also, one-third of euthanised 2- to 3-year-old thoroughbred racehorses were diagnosed post mortem with metacarpophalangeal joint arthritis [2]. Intra-articular (IA) injections of glucocorticoids are routinely used in the treatment of equine joint disease. Both clinical studies and experimental studies demonstrate the effectiveness of glucocorticoids in the inflamed joint [3–7]. The effect of dexamethasone administered both intravenously (IV) and IA has been investigated after lipopolysaccharide (LPS) challenge of the equine joint using total protein concentration in synovial fluid as response biomarker [5]. However, that study only compared the effect after IV and IA administration and did not report any dexamethasone plasma nor synovial fluid concentrations of dexamethasone. Synovial fluid concentrations and plasma disposition has been described for several glucocorticoids after IA administration to healthy joints [8–17]. This quantitative information might also be used in anti-doping and medication control to assess irrelevant drug concentrations, which protect both animal welfare and the integrity of racing and equestrian sports [18, 19]. However, the disposition of dexamethasone in synovial fluid from inflamed joint has not been reported. The aim of this study was to investigate and describe the synovial fluid and plasma dexamethasone concentration over time and to explore the relation between synovial fluid concentration and response using clinical endpoints as response biomarkers after IA injection of dexamethasone disodium salt solution in an equine model of synovitis.

## Methods

### Animals

Three Standardbred mares and three Standardbred geldings (horses A–F), 3–9 years old and weighing 429–550 kg, were included in the study. Immediately before collection of baseline data no signs of radiocarpal joint inflammation (joint effusion, heat) was detected upon clinical examination and front leg lameness was 1.5 or less (American Association of Equine Practitioners lameness scale) during baseline collection.

### Experimental design and intra-articular injections

This study was prospective, randomised, placebo-controlled and blinded. Three days before treatment, the horses were transported to the study location for acclimatisation. The horses were assigned to an experiment including two treatments, LPS + dexamethasone 21-phosphate disodium salt solution (DSP, Dexadreson, 2 mg/mL, Intervet AB, Sollentuna, Sweden) and for control LPS + saline was administered in the contralateral joint. Dexamethasone dose and treatment regimen is shown in Table 1.

**Table 1:** Dose and treatment regimen

Horse	Dose (mg)	Treatment #1	Limb	Treatment #2	Limb
A	0.3	LPS + dexamethasone	RF	LPS + saline	LF
B	0.1	LPS + saline	LF	LPS + dexamethasone	RF
C	0.03	LPS + saline	RF	LPS + dexamethasone	LF
D	1	LPS + saline	LF	LPS + dexamethasone	RF
E	3	LPS + saline	RF	LPS + dexamethasone	LF
F	0.01	LPS + dexamethasone	LF	LPS + saline	RF

*LF* left front, *RF* right front

During experimental periods, the horses were kept in individual boxes and fed hay and concentrate (Champion komplett, Felleskjøpet, Lillestrøm, Norway). Water was available ad libitum. During wash-out periods the horses were on pasture. Before treatment, the hair over the jugular vein was clipped and the skin was disinfected with chlorhexidine solution and 70% ethanol. One 2.1 × 130 mm intravenous catheter (MILA International Inc., Florence, KY, USA) was placed in the jugular vein and secured with three sutures. Before arthrocentesis, the hair over the radiocarpal joint was clipped and the skin was disinfected with chlorhexidine solution and 70% ethanol.

Inflammation was induced in the radiocarpal joint by means of LPS from *Escherichia coli* 055:B5 purified by phenol extraction (Product Number L2880) (Sigma-Aldrich). A stock solution was prepared by diluting the LPS in sterile water. In a second step, the LPS was diluted in sterile physiological saline to a concentration of 1 ng/mL. Aliquots were frozen at − 70 °C in siliconised tubes, thawed and vortexed for 15 min immediately before use, according to the manufacturer’s instructions. A total volume of 2 mL LPS solution was injected into the joint at time 0 using a glass syringe (Hamilton Company, Timis, Romania). Two hours after LPS injection, equivalent volumes (2 mL) of either 0.9% saline or DSP diluted in saline were injected into the joint. All joint injections and samplings were performed using 0.8 × 35 mm needles. Each of the six horses was treated with one dose dexamethasone injected once into the radiocarpal joint during LPS + DSP treatment. A minimum of 3 weeks wash-out period was applied between treatments. A nose twitch was applied before injections and collection of synovial fluid in order to restrict the horse from sudden movements. The study was approved by the Norwegian Animal Research Authority (Forsøksdyrutvalget 2013/61618-1).

### Sampling protocol

Synovial fluid samples and plasma samples for drug concentration determination were collected at time − 2 (before LPS injection) and 0 (before DSP or saline injection), 2, 4, 8, 22, 26, 30, 46, 50, 54, 70 and 74 h after DSP and saline injection. Three additional blood samples were also drawn at 5, 20 and 40 min after DSP and saline injection. Immediately before injection or sampling the skin over the joint was desensitised by means of a cooling spray (Articare Cold Spray, BSN Medical Ltd. Hull, UK). Blood and synovia were centrifuged at 1800*g* for 10 and 5 min, respectively. The supernatant was transferred to microcentrifuge tubes and immediately frozen at − 20 °C, moved within 24 h and stored at − 70 °C pending analysis.

Clinical endpoints (CEs) baseline was recorded three times, typically between 15.00 and 18.00 the day before LPS challenge. CEs were also recorded before collection of synovial fluid and at one additional occasion 6 h after dexamethasone administration. The CEs were: rectal temperature (RT) measured by means of a digital thermometer, local skin temperature (ST) measured by means of a digital infrared thermometer (Fluke 574 cf, SR Automation AS, Asker, Norway) and joint-circumference (JC) measured by means of a measuring-tape. Lameness was evaluated in trot (8 × 20 m in a straight line on flat concrete floor indoors). Lameness was subjectively scored by three experienced clinicians using the American Association of Equine Practitioners (AAEP) lameness scale. Objective lameness analysis was performed by means of a commercial inertial measure unit based gait analysis system (Lameness Locator^®^, Equinosis, St. Louis, MO, USA) for lameness detection [20]. Two uni-axial accelerometer was used. One was mounted to a head bumper attached to the bridle over the poll and one was taped to the midline of the pelvis at the level of the tubera sacrale. One uni-axial gyroscope was attached dorsally to the proximal and middle phalanges of the right forelimb for stride segmentation. Vertical uni-axial acceleration was recorded at 200 Hz and data were transmitted wirelessly from the sensors to a computer running the data collection software. Objective motion data were processed with the software package for the gait analysis system. Raw uni-axial acceleration signals from head and pelvis sensors, aligned with the global vertical axis in the standing position, were first transformed into displacement signals using a custom-designed, error-correcting, double-integration technique and the signal from the right forelimb gyroscope [20]. From the displacement signal local minima of the head were identified (two per stride). Forelimb impact lameness was measured by means of differences between the two local minima (HD_min_) during left and right stance phases, computed for each stride. Results are reported as change in absolute HD_min_ values from baseline.

### Dexamethasone IV-data

Dexamethasone plasma concentration–time data from a previous study [21] was used in the pharmacokinetic analyses in order to increase the data-set. In that study six Standardbred horses (four mares and two geldings) 6–20 years old and weighing 430–584 kg were used in a randomized crossover design including three treatments with dexamethasone at various doses. Each treatment started with an intravenous bolus dose immediately followed by 3 h of constant rate infusion of dexamethasone 21-phosphate disodium salt (Dexadreson 2 mg/mL, Intervet AB, Sollentuna, Sweden). The dose levels were (bolus + infusion) 0.1 + 0.07 μg/kg, 1 + 0.7 μg/kg and 10 + 7 μg/kg dexamethasone. Before the bolus dose (time = 0) a pre-dose blood sample was drawn. Additional blood samples were drawn during and after infusion at hours 1, 2, 3, 4, 5, 6, 9, 12, 18, 24, 36 and 48. A minimum of a 1-week wash-out period was allowed between drug treatments.

### Dexamethasone quantification

Plasma and synovial fluid dexamethasone concentrations were analysed and quantified with the use of ultra high performance liquid chromatography–tandem mass spectrometry (UHPLC–MS/MS). Dexamethasone reference compound was acquired from Toronto Research Chemicals (North York, ON, Canada) and the internal standard ^2^H_4_-dexamethasone (dexamethasone-d4) was purchased from CDN Isotopes through QMX Laboratories Ltd. (Essex, UK). The water was purified using a Milli-Q water purification system (Millipore, Bedford, MA, USA). All other chemicals were of analytical grade or better and used without further purification. Synovial fluid from control treatment was used as blank matrix for preparations of calibrators and control samples (QC).

Samples were pretreated by diluting each synovial sample (50 µL) with 950 µL of aqueous potassium phosphate buffer (0.1 M, pH 6.05). To 100 µL of the diluted sample, 50 µL of the internal standard (dexamethasone-d4, 0.05 µg/mL), 500 µL of potassium phosphate buffer (0.1 M, pH 6.05) were added. Liquid–liquid extraction was carried out to 2.0 mL tert-butylmethylether by vortex mixing for 5 min and subsequent centrifugation for 10 min. To facilitate phase separation, the samples were then frozen at − 70 °C for 30 min after which the organic phase was decanted to another tube and evaporated under a gentle stream of air at 55 °C. The samples were reconstituted in 100 µL of aqueous formic acid (0.1%)/methanol (1:1 v/v) and transferred to vials for UHPLC–MS/MS analysis.

The extracts were analyzed with UHPLC–MS/MS. A Waters Acquity UPLC system was coupled to a Quattro Ultima Pt tandem quadrupole mass spectrometer with an electrospray interface operating in the positive mode (Waters Corporation, Milford, MA). The column was an Acquity UPLC BEH C18 (length 100 mm, I.D. 2.1 mm, particle size 1.7 µm) from Waters Corporation kept at 65 °C. The mobile phase consisted of (A) ammonium acetate (aq, 2.0 mM) and (B) formic acid (0.1%) in acetonitrile. A gradient was run as follows: initially 23% B for 4.7 min, 23–90% B in 0.1 min, constant at 90% B for 1.0 min, 90–23% B in 0.1 min, constant at 23% B for 0.9 min. The total run time was 6.8 min, the flow rate was 650 µL/min and the injection volume was 10 µL.

The mass spectrometric settings were as follows: a positive capillary voltage of 3.5 kV, the desolvation and source block temperatures were 300 °C and 120 °C and the cone and desolvation gas flows were 121 and 830 L/h, respectively. The quantifications were performed in the selected reaction monitoring (SRM) mode with the collision cell filled with argon gas at a pressure of 2.4 × 10^−3^ mBar. The transitions used in SRM were *m*/*z* 393 → 355 for dexamethasone (collision energy 14 eV, cone voltage 50 V) and *m*/*z* 397 → 359 for dexamethasone-d4 (collision energy 14 eV, cone voltage 50 V). The dwell time was 0.01 s.

Stock solutions of dexamethasone, and the internal standard were prepared in methanol at approximately 0.3 mg/mL. These solutions were diluted and used to spike blank synovial fluid to obtain calibration and QC samples. The calibration was performed by linear curve fit (weighting factor of 1/x^2^) of the peak area ratio (analyte/internal standard) as a function of the analyte concentration. The LLOQ for dexamethasone in synovial fluid was 0.38 ng/mL. The precision expressed as the relative standard deviation (RSD) in the results of quality control samples in synovia was in the interval 2.7–7.8% and the accuracy was in the range of 97–109%.

The analytical method for dexamethasone in plasma is further described in our previous work [22]. The lower limit of quantification (LLOQ) for dexamethasone in plasma was 0.025 ng/mL.

### Dexamethasone concentration–time analyses

**Fig. 1:**
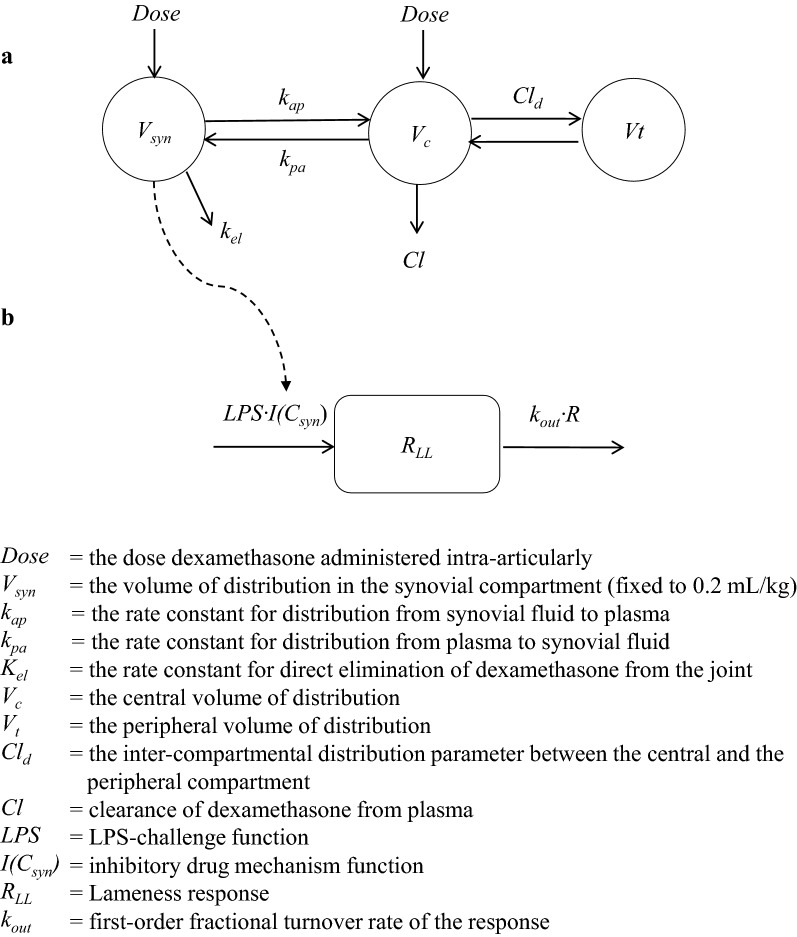
Illustration of the sequential approach that was adopted to estimate the PK/PD parameters. The upper row shows the dexamethasone synovial fluid and plasma disposition model (**a**) and the lower row the pharmacodynamic model (**b**) describing the LPS challenge and lameness response (*R*_*LL*_). The post hoc estimates of individual PK parameters were fixed to their post hoc estimate to “drive” the drug-mechanism function (*I*(*C*_*syn*_)) acting on production of lameness response induced by the LPS-challenge [25]

A non-linear mixed effects (NLME) approach that is the appropriate tool [23] to analyse unbalanced data set obtained in different trials was used to estimate pharmacokinetic and pharmacodynamic parameters. A comprehensive three compartment model (Fig. 1) was fitted to the dexamethasone synovial fluid concentration–time data from the six horses in the current study and to the dexamethasone plasma concentration–time data in the current study and from the study in [21]. The disposition of dexamethasone in synovial fluid was described as:

1 $$\frac{{dAmt_{syn} }}{{d_{t} }} = \left[ {Amt_{p} \cdot k_{pa} - Amt_{syn} \cdot k_{ap} \left] - \right[Amt_{syn} \cdot k_{el} } \right]$$

where *Amt*_*syn*_ and *Amt*_*p*_ are the dexamethasone amount in synovial fluid and in plasma, respectively. The *k*_*pa*_, *k*_*ap*_ and *k*_*el*_ are the first-order rate constants from plasma to synovial fluid, from synovial fluid to plasma and for direct elimination from the joint, respectively. The dexamethasone synovial fluid concentration (*C*_*syn*_) was obtained by:

2 $$C_{syn} = \frac{{Amt_{syn} }}{{V_{syn} }}$$

For identifiability reason, the average volume of the synovial compartment (*V*_*syn*_) was fixed at 0.2 mL/kg and the individual value for each horse (*V*_*horse*_) was obtained by:

3 $$V_{horse} = 0.2 \cdot e^{{\eta_{horse} }}$$

where η_*horse*_ is the deviation of each horse from its (fixed) typical value.

The disposition of dexamethasone in the central compartment after IA administration of dexamethasone was described as:

4 $$  \begin{aligned} \frac{{dAmt_{p} }}{dt} &= - \left[ {Cl \cdot C_{p} } \right] - Cl_{d} \cdot \left[ {C_{p} - C_{t} }\right] \\&\quad- \left[{Amt_{p} \cdot k_{pa} - Amt_{syn} \cdot k_{ap} } \right] \end{aligned} $$

where *C*_*p*_ and *C*_*t*_ denote the dexamethasone concentration in synovial fluid, the central and peripheral compartment, respectively. *Amt*_*syn*_, *k*_*pa*_ and *k*_*ap*_ as defined in Eq. (1), *Cl* is the plasma clearance of dexamethasone and *Cl*_*d*_ is the inter-compartmental distribution. Dexamethasone disposition in the central compartment after IV administration of dexamethasone was described as:

5 $$\frac{{dAmt_{p} }}{dt} = - \left[ {Cl \cdot C_{p} } \right] - Cl_{d} \cdot \left[ {C_{p} - C_{t} } \right]$$

and the dexamethasone amount in the peripheral compartment *Amt*_*t*_ was described as:

6 $$Amt_{t} = Cl_{d} \cdot \left( {C_{p} - C_{t} } \right)$$

where *C*_*p*_ and *C*_*t*_ were obtained by Eqs. (7) and (8):

7 $$C_{p} = \frac{{Amt_{p} }}{{V_{c} }}$$

8 $$C_{t} = \frac{{Amt_{t} }}{{V_{t} }}$$

where *V*_*c*_ is the central volume of distribution, *V*_*t*_ is the peripheral volume of distribution.

The between subject variability (BSV) was described using an exponential model of the form:

9 $$\theta_{parameter\_i} = \theta_{tv\_parameter} \cdot e^{\eta }$$

where *θ*_*parameter_i*_ is the value of theta for respective parameter in the ith horse, $\theta_{tv\_parameter}$ is the typical population value of the parameter (e.g. *V*_*c*_, *V*_*t*_, *Cl*, *Cl*_*d*_ etc.) and $\eta_{i}$ is the deviation from the corresponding theta population value associated to the *i*th horse. The exponential model assumes a log-normal distribution of parameters, i.e. that the distribution of the *etas* ($\eta_{i}$) is normal in the log-domain, with a mean of 0 and a variance ω^2^ where:

10 $$\eta \approx N\left[ {0,\omega^{2} } \right]$$

Shrinkage of random effects toward the means according to Karlsson and Savic [24] was described as:

11 $$shrinkage = 1 - \frac{{SD\left( {EBE_{\eta } } \right)}}{\omega }$$

where *SD*(*EBEη*) is the standard deviation of the individual values of the Empirical Bayesian Estimates (EBE) of *η*.

When the three compartment model was fitted to IV-data from the study in [21], clearance was assumed to possibly vary with dose and was considered as a covariate described as:

12 $$Cl = \theta_{tvCl} \cdot \left[ {1 + \theta_{1} dose_{level} } \right]$$

where *θ*_*tv*_, *θ*_*1*_ and *dose*_*level*_ are the typical value for clearance for a theoretical dose of 0, *θ*_*1*_ reflect the change in clearance by dose level and the dose level, respectively.

From the model parameters following secondary parameters were derived:

The terminal slope (*β*_*p*_) of the dexamethasone plasma concentration–time course was described as:

13 $$ \begin{aligned} \beta_{p} & = 0.5 \cdot \left[ {\frac{{Cl_{d} }}{{V_{c} }} + \frac{{Cl_{d} }}{{V_{t} }} + \frac{Cl}{{V_{c} }} - \left[ {\left( {\frac{{Cl_{d} }}{{V_{c} }} + \frac{{Cl_{d} }}{{V_{t} }} + \frac{Cl}{{V_{c} }}} \right)^{2} } \right.} \right. \\ & \quad \quad \left. {\left. { - 4\frac{{Cl_{d} }}{{V_{t} }} \cdot \frac{Cl}{{V_{c} }}} \right]^{0.5} } \right] \\ \end{aligned} $$

The initial slope (α_p_) of the plasma dexamethasone concentration–time course was described as:

14 $$\alpha_{p} = \frac{{Cl_{d} }}{{V_{t} }} \cdot \frac{{\frac{Cl}{{V_{c} }}}}{\beta }$$

The terminal half-life (*t*_*1/2β*_) of the plasma dexamethasone concentration–time course was described as:

15 $$t_{1/2\beta } = \frac{ln2}{\beta }$$

The initial half-life (*t*_*1/2α*_) of the plasma dexamethasone concentration–time courses was described as:

16 $$t_{1/2\alpha } = \frac{ln2}{ \propto }$$

### LPS-challenge model and lameness response analyses

The LPS synovial concentration was not known and the time course of the challenge function (*S*_*LPS*_) was described as:

17 $$ \begin{aligned} S_{LPS} & = \left[ {A_{LPS1} \cdot k_{LPS1} \cdot t \cdot e^{{ - k_{LPS1} \cdot t}} } \right]^{\gamma 1} \\ & \quad + \left[ {A_{LPS2} \cdot k_{LPS2} \cdot t \cdot e^{{ - k_{LPS2} \cdot t}} } \right]^{\gamma 2} \\ \end{aligned} $$

where *A*_*LPS1*_, *A*_*LPS2*_, *k*_*LPS1*_ and *k*_*LPS2*_ represent the maximal input rates and the rate constant controlling the time development of the challenge function. The gamma (γ_1_, γ_2_) parameters are exponents amplifying the response to the challenge. The rate of change $\left( {\frac{dR}{dt}} \right)$ of lameness response measured by means of Lameness locator^®^ was then described as follows:

18 $$\frac{dR}{dt} = S_{LPS} - k_{out} \cdot R$$

where *k*_*out*_ and *R* represent the fractional turnover rate of response and the lameness response.

Dexamethasone was assumed to inhibit lameness response to the LPS challenge. A sequential approach was adopted to estimate the PD parameters. The post hoc estimates of individual PK parameters were fixed to their post hoc estimate to ‘drive’ the inhibitory function [25]. The inhibitory function (*I*(*C*_*s*_)) was described as:

19 $$I\left( {C_{s} } \right) = 1 - \frac{{I_{max} \cdot C_{syn} }}{{IC_{50} + C_{syn} }}$$

where *I*_*max*_ and *IC*_*50*_ are the maximum drug-induced inhibition of lameness response and the dexamethasone synovial fluid concentration at 50% reduction in the lameness response, respectively.

The drug mechanism function was then incorporated in Eq. (16) to give Eq. (18):

20 $$\frac{dR}{dt} = S_{LPS} \cdot I\left( {C_{s} } \right) - k_{out} \cdot R$$

Phoenix NLME version 8.0 (Certara, St. Louis, MO, USA) was used in regression of data. A multiplicative (proportional) error (weighting) model was used for weighting dexamethasone-time data and lameness response-time data.

### Statistics

Independently of the PK/PD-modelling approach the data was subjected to conventional statistical hypothesis testing by means of a linear mixed-effects model. Time and dose were used as categorical fixed effects and horse as random effect. Data was compared between doses for every time-point. An ad hoc analysis (Dunnett’s test) was performed in order to compare data after LPS administration with the pre-administration data. The repeated measures structure of the data was accounted for by including an autoregressive correlation structure on the error term within each individual. Statistical significance was considered when P < 0.05. All statistical analyses were performed using the statistical software R version 3.4.4 (The R Foundation for Statistical Computing, Vienna, Austria).

## Results

### Disposition of dexamethasone

**Fig. 2:**
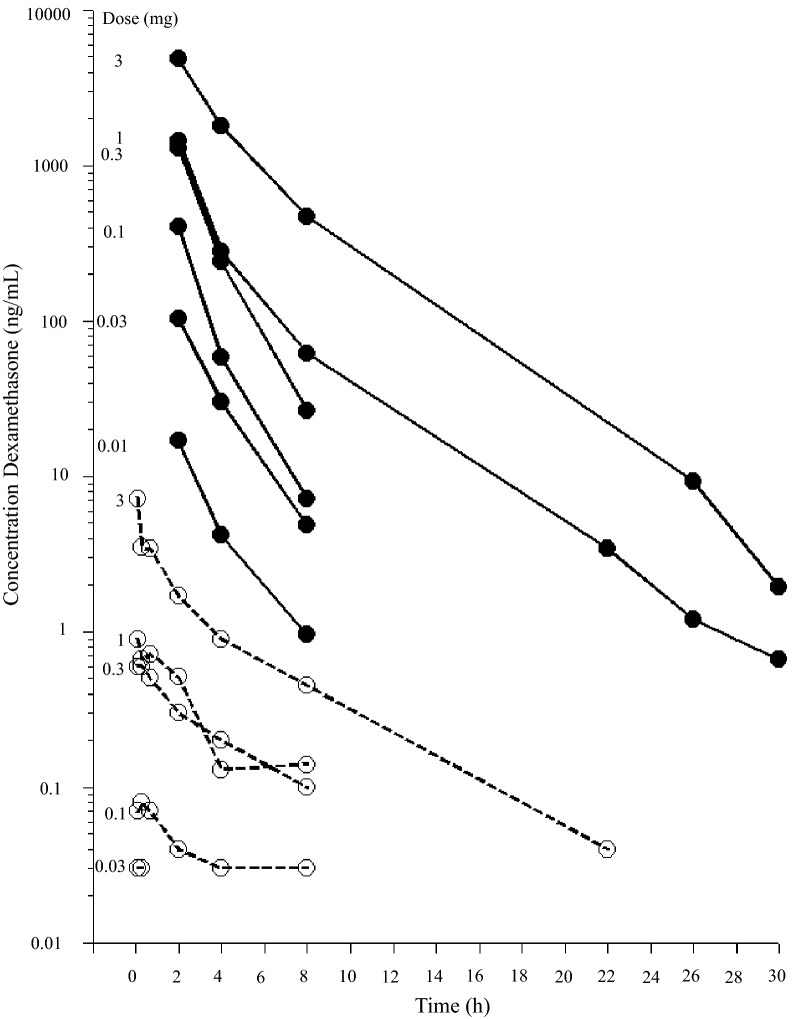
Observed dexamethasone synovial fluid (filled circles) and plasma (open circles) concentrations over time after intra articular administration of dexamethasone sodium phosphate into the LPS-challenged joint

In the horses receiving the four lower doses, dexamethasone was quantifiable (LLOQ 0.38 ng/mL) in synovial fluid samples collected 2, 4 and 8 h after injection. In the horses receiving the two higher doses, dexamethasone was quantifiable up to 30 h (six samples) after injection (Fig. 2).

For horse F that received 0.01 mg dexamethasone IA no dexamethasone plasma concentrations were quantifiable (LLOQ 0.025 ng/mL). For horse C that received 0.03 mg, dexamethasone was only quantifiable in the samples collected 5 and 20 min after DSP injection (both 0.03 ng/mL). In the horses receiving the four higher doses (Table 1) dexamethasone was quantifiable up to 24 h (5–7 plasma samples) and maximum concentration was observed within 40 min (Fig. 2).

**Fig. 3:**
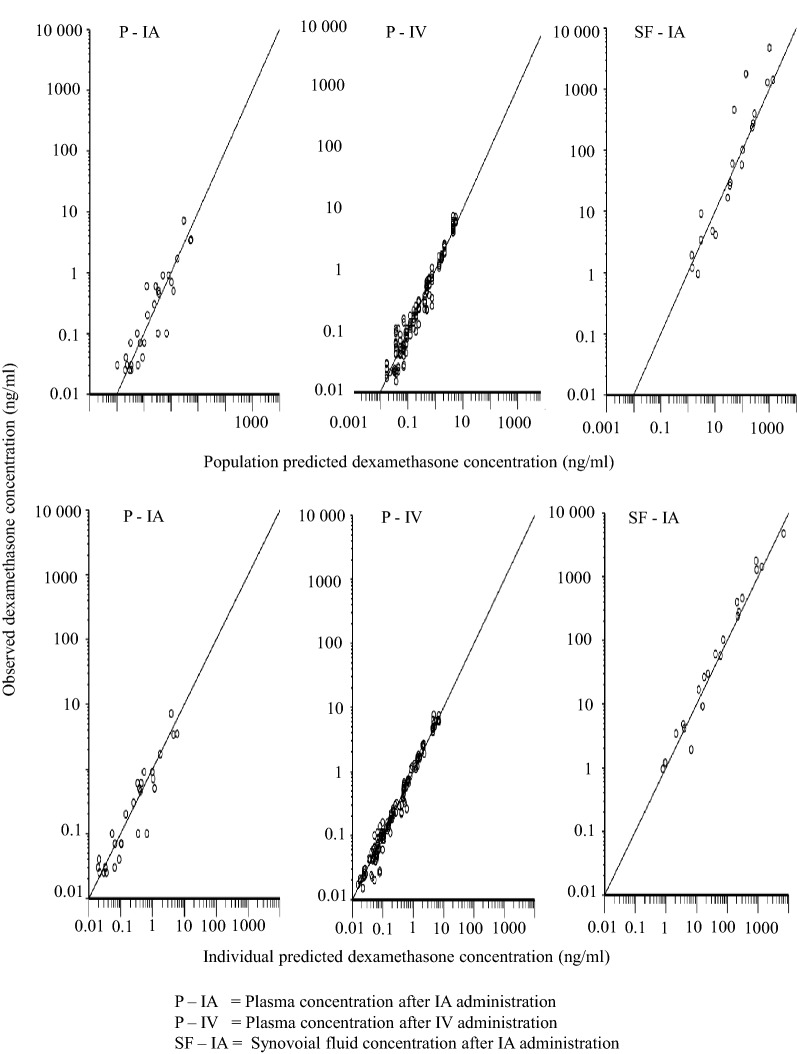
Observed dexamethasone concentration vs population model predicted dexamethasone concentration (upper row) and observed dexamethasone concentration vs individual model predicted dexamethasone concentration (lower row)

**Fig. 4:**
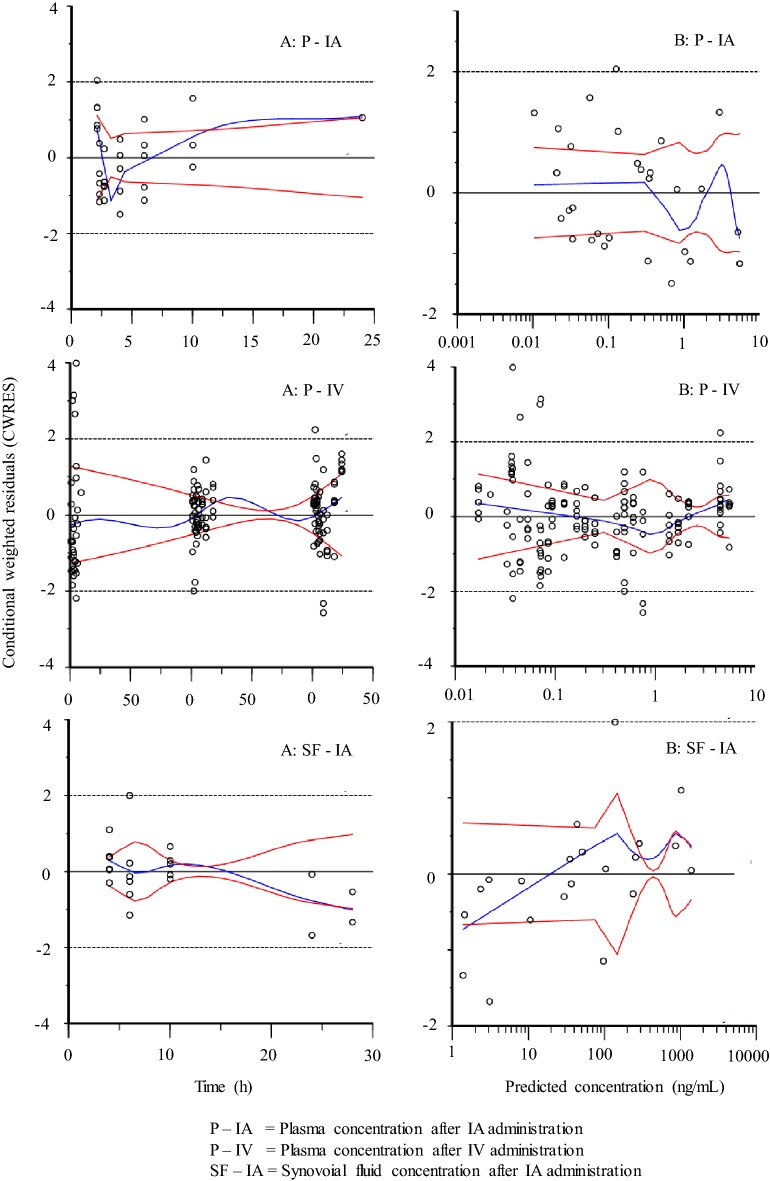
Conditional weighted residuals (CWRES) over time (left column, **a**) and CWRES over population model predictions (right column, **b**) for dexamethasone in plasma (upper row) and synovial fluid (lower row) after treatment with LPS + dexamethasone sodium phosphate administered intra-articularly and in plasma (intermediate row) after treatment with dexamethasone sodium phosphate administered intra-intravenously

A three-compartment model (including one compartment for synovial fluid) was fitted to experimental dexamethasone synovial fluid data and dexamethasone plasma data from all horses in this study and from literature plasma raw data collected from [21]. The model estimated and derived pharmacokinetic parameters from the horses in this study are shown in Table 2. Experimental dexamethasone data were evenly distributed around the line of unity for both the structural and the random component of the three compartment model (Fig. 3). The conditional weighted residuals (CWRES) over time were concentrated between y = − 2 and y = + 2 for dexamethasone plasma and synovial fluid concentrations (Fig. 4). The typical values for disposition in synovial fluid and plasma are given in Table 2. The typical value for total plasma clearance of dexamethasone was 246 mL/kg/h. For literature data from [21], dexamethasone was administered in three doses. Hence, dose was used as a covariate and clearance was 426 mL/kg/h and 428 mL/kg/h after administration of the intermediate and the high dose. The *t*_*1/2α*_ and *t*_*1/2β*_ in plasma was 0.6 h and 4.3 h. The fraction of the administered dose that was eliminated directly from the joint without gaining access to plasma for the first 48 h after administration was 0.5–2.1%.

**Table 2:** Model typical values (tv) and the individual pharmacokinetic parameter estimates for the horses A–F

Horse	*Dose* (mg)	*V*_*syn*_ (mL/kg)	*k*_*ap*_ (1/h)	*k*_*pa*_ (1/h)	*k*_*el*_ (1/h)	*V*_*c*_ (mL/kg)	*V*_*t*_ (mL/kg)	*Cl* (mL/kg/h)	*Cl*_*d*_ (mL/kg/h)
*tv*	na	0.2	0.6	0.15	0.010	338	1123	246	495
F	0.01	0.49	0.61	0.15	0.010	279	1135	247	491
C	0.03	0.27	0.61	0.14	0.011	148	1114	253	690
B	0.1	0.26	0.65	0.14	0.013	126	1018	266	778
A	0.3	0.18	0.74	0.15	0.009	85	997	417	598
D	1	0.20	1.06	0.15	0.010	308	1133	446	637
E	3	0.03	1.91	0.15	0.010	230	1263	470	763

Where *tv* is the typical value for the population, *dose* is the amount dexamethasone administered into the joint, *V*_*syn*_, *V*_*c*_ and *V*_*t*_ are the volume of distribution in the synovial compartment (typical value was fixed to 0.2 mL/kg), the central compartment and the peripheral compartment, respectively. *Cl* is the plasma clearance and *Cl*_*d*_ is the inter-compartmental distribution parameter. *k*_*ap*_, *k*_*pa*_ and *k*_*el*_ are the rate constants from the joint to the plasma, the plasma to the joint and direct elimination from the joint, respectively. Note that shrinkage was high (> 0.3) for *k*_*ap*_*, k*_*pa*_ *k*_*el*_, *V*_*syn*_ and *Cl* due to a poor estimate of the random component of the model for these parameters and corresponding post hoc values were shrinked toward their population values

**Fig. 5:**
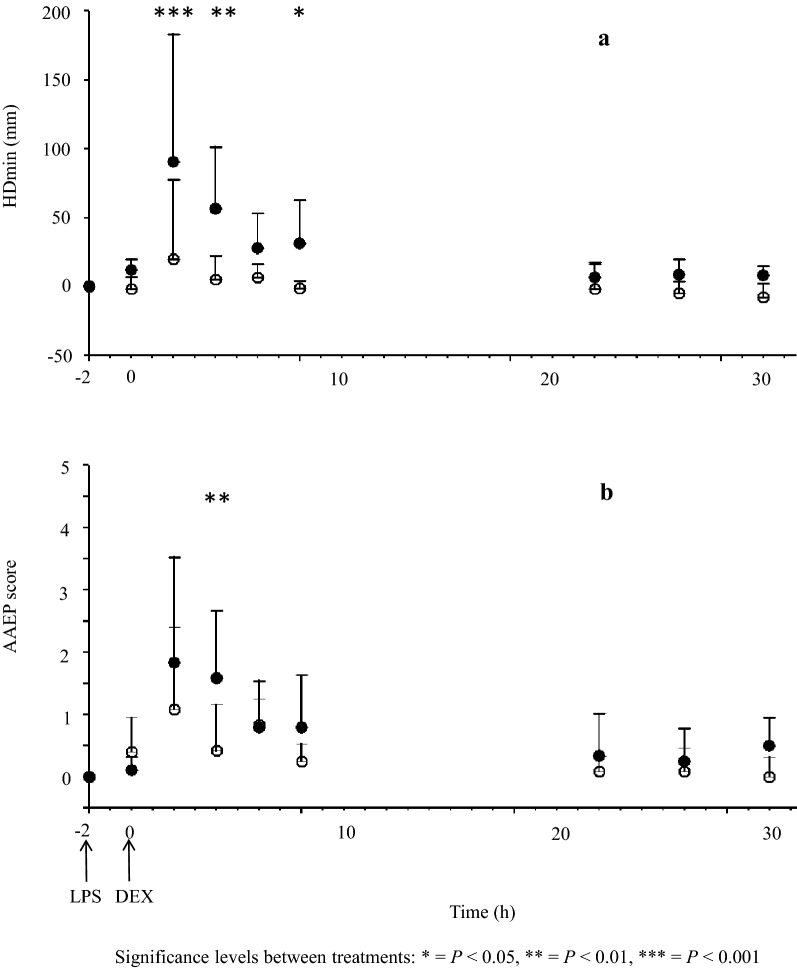
Change in (mean and standard deviation) lameness relative to baseline in six horses challenged with 2 ng lipopolysaccharides in the radiocarpal joint at hour − 2. At hour 0 either saline (filled circles) or dexamethasone (open circles) was injected in the LPS challenged joint. The horses received one dose dexamethasone sodium phosphate each. The doses were 0.01, 0.03, 0.1, 0.3, 1, and 3 mg administered in an injection volume of 2 mL. Upper plot (**a**): change in minimum head height differences (HD_min_) scored objectively. Lower pot (**b**): change in American Association of Equine Practitioners (AAEP) lameness score

There were no differences in clinical endpoint baseline between the two treatment protocols. The LPS + saline treatment increased objective lameness score using objective (HD_min_) lameness data compared to baseline at 4 h (P < 0.001) and 6 h (P < 0.001) after LPS-administration. The LPS + DSP treatment did not increase lameness score compared to baseline using HD_min_ data. After treatment with LPS + DSP the horses were less lame at 4 h (P 0.001), 6 h (P= 0.004) and 10 h (P = 0.04) compared to treatment with LPS + saline (Fig. 5a). The LPS + saline treatment increased subjective lameness score compared to baseline at 4 h (P < 0.001) and 6 h (P < 0.001) after LPS-administration. The LPS + DSP treatment increased subjective lameness score at 4 h (P = 0.008) compared to baseline. After treatment with LPS + DSP horses were less lame at 6 h (P = 0.008), compared to treatment with LPS + saline (Fig. 5b).

There was no difference in JC and ST between the two treatments. For RT no difference between the two treatments was observed from 0 to 72 h. At 76 h RT was higher in the LPS + saline group (P = 0.03). No signs of septic arthritis were observed during or in the following days after LPS injection and sample collection.

**Fig. 6:**
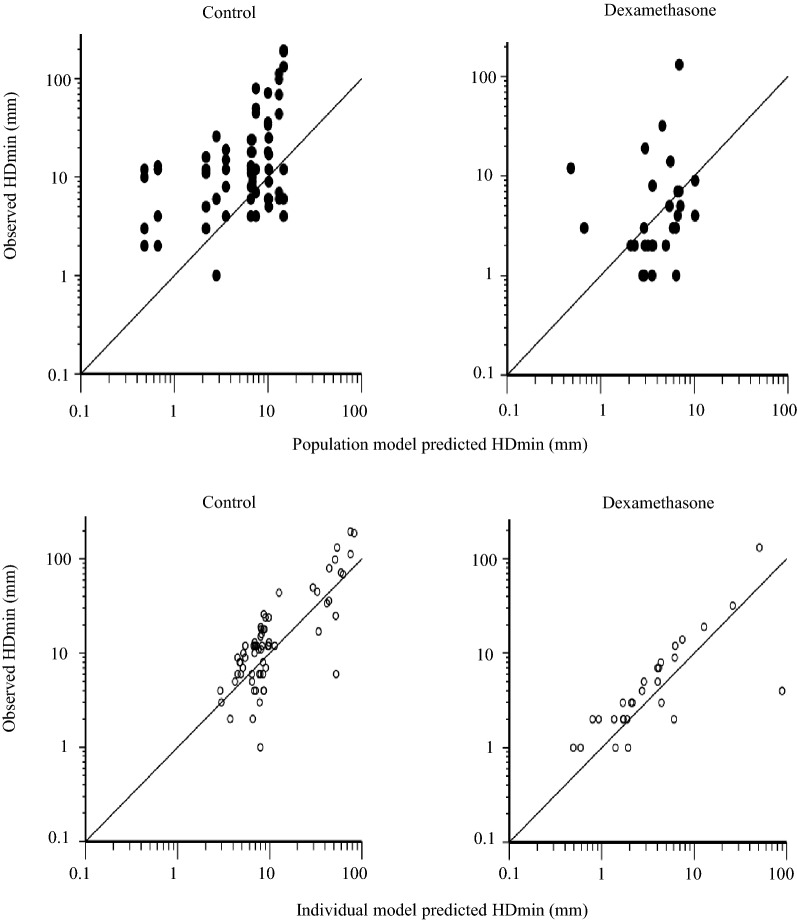
Observed lameness response (HD_min_) vs population model predicted HD_min_ (upper row) and observed HD_min_ vs individual model predicted HD_min_ (lower row) after treatment with either LPS + saline (left column) or LPS + dexamethasone sodium phosphate (right column) administered intra-articularly

**Fig. 7:**
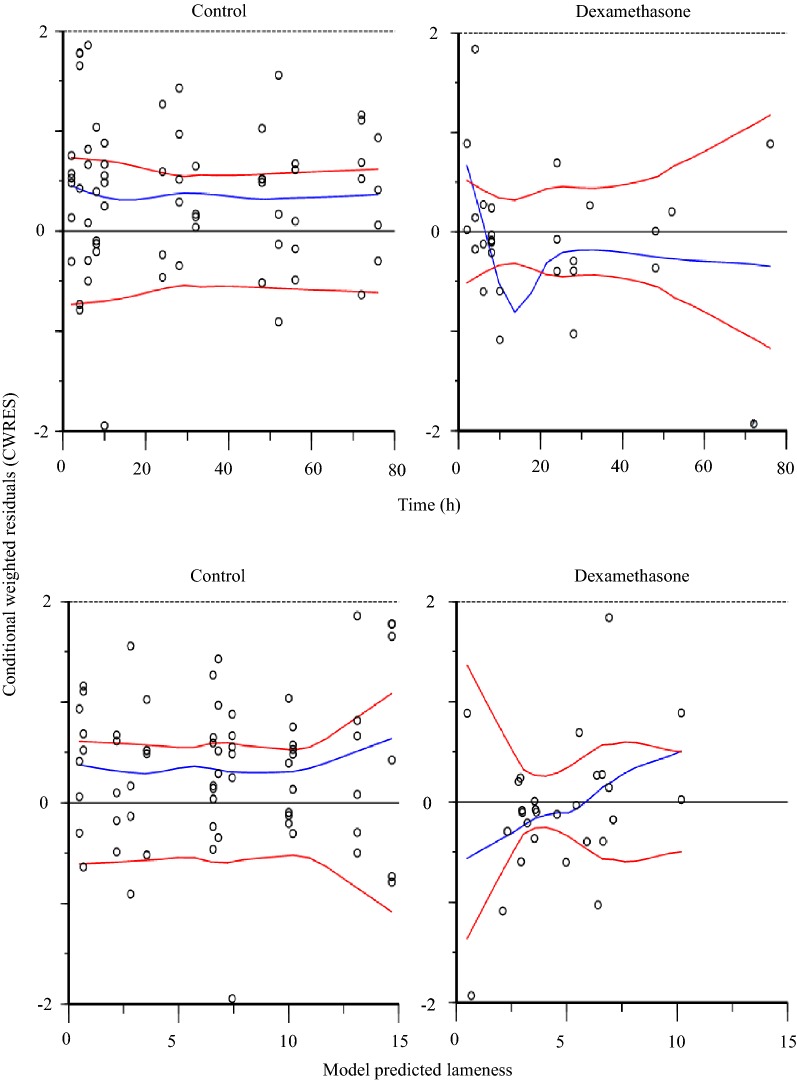
Conditional weighted residuals (CWRES) over time (upper row) for and CWRES versus lameness response (lower row) after treatment with either LPS + saline (left column) or LPS + dexamethasone sodium phosphate (right column) administered intra-articularly

Subjective and objective (HD_min_) lameness scores are shown in Fig. 5. The LPS-challenge + intra articular saline induced an increase in HD_min_ (indicating impact lameness in the induced limb) in all horses compared to baseline. Median (range) HD_min_ was 96 mm (12–196). The maximum HD_min_ was observed within 8 h in all horses. The HD_min_ score then returned to baseline within 24 h after LPS-challenge in five horses and within 32 h in one horse. A pharmacodynamic model was fitted to experimental lameness response data from all horses. Experimental HD_min_ data were evenly distributed around the line of unity for both the structural and the random component of the pharmacodynamics model (Fig. 6). CWRES over time were concentrated between y = − 2 and y = + 2 for lameness data (Fig. 7). The model estimated typical values are shown in Table 3

**Table 3:** Model typical values (tv) and the individual pharmacodynamic parameter estimates for the horses A–F

Horse	Dose (mg)	*IC*_50_ (ng/mL)	*I*_*max*_	*k*_*out*_ (1/h)	*A*_*LPS1*_ (*R*/h)	*A*_*LPS2*_ (*R*/h)	*k*_*LPS1*_ (1/h)	*k*_*LPS2*_ (1/h)	*γ*_1_	*γ*_2_
*tv*	na	4.0	0.84	0.44	3.5	48.4	0.04	0.65	3.9	0.8
F	0.01	3.4	0.85	0.39	3.4	41.2	0.07	0.68	2.2	0.6
C	0.03	2.9	0.85	0.59	3.4	43.3	0.06	0.68	1.6	0.6
B	0.1	3.4	0.86	0.35	3.4	40.9	0.05	0.68	4.9	0.5
A	0.3	3.1	0.85	0.39	3.4	47.5	0.007	0.69	12.8	1.5
D	1	3.4	0.84	0.33	3.4	41.7	0.08	0.67	4.0	0.6
E	3	3.9	0.85	0.39	3.4	41.2	0.07	0.68	2.1	0.6

Where *tv* is the typical value for the population, *dose* is the amount dexamethasone administered into the joint, *IC*_50_, *I*_*max*_ and *k*_*out*_ are the potency value, the efficacy value (with a maximal possible value of 1) and fractional elimination rate of the response, respectively. *A*_*LPS1*_, *A*_*LPS2*_, *k*_*LPS1*_, *k*_*LPS2*_, *γ*_*1*_ and *γ*_*2*_ are maximal input rates, the rate constant controlling the time development of the challenge function and exponents shaping (amplifying) the response to the LPS-challenge, respectively. Note that shrinkage was high (> 0.3) for *IC*_*50*_*, I*_*max*_, *A*_*LPS1*_, *A*_*LPS2*_ and *k*_*LPS2*_ due to a poor estimate of the random component of the model for these parameters and corresponding post hoc values were shrinked toward their population values

## Discussion

This study investigated and described dexamethasone disposition in synovial fluid and plasma after IA administration of DSP in the inflamed equine joint. Simultaneously it reports the relation between synovial fluid and plasma concentration and CE response to IA dexamethasone treatment. The present data were analysed with a NLME model (also known as population modelling) consistent with the U.S Food and Drug Administration guidelines [23]. The population PK approach allows the analysis of data from a variety of unbalanced designs as well as from studies that are normally excluded because they do not lend themselves to the usual forms of pharmacokinetic analysis, such as concentration data obtained from paediatric and elderly patients, or data obtained during the evaluation of the relationships between dose or concentration and efficacy or safety [26]. For our data sets, a classical two-stages analysis led to the biased estimate of some parameters as the *IC*_50_ due to outlying individual estimate whereas the NLME-analyses allowed the estimate of robust typical (here synonym of mean) values of these parameters even if the between subject variability was not properly estimated for some parameters from our limited number of horses. This approach is also recommended in toxicokinetic trials for which a limited number of subject are often investigated using different unbalanced and sparsely sampling design [27, 28].

The pharmacokinetic model fitted to experimental synovial fluid dexamethasone data satisfactory mimicked the data and no major bias or model misspecifications were suspected (Figs. 3 and 4). Quantitative information about synovial fluid and plasma disposition of dexamethasone was provided (Table 2). To our knowledge, the dexamethasone concentration–time course in synovial fluid has not previously been reported. In plasma, the total clearance, the central and peripheral volume of distribution and the initial and terminal half-lives were consistent with the literature [8, 21, 29, 30]. Dexamethasone was removed from the joint when synovial fluid was collected and possibly by enzymatic breakdown in the joint. The cumulative amount directly removed from the joint was 0.5–2.1% of the administered dose. This a suggest a systemic bioavailability around 98–99.5% for dexamethasone after IA injection in the inflamed joint, which is consistent with the bioavailability of dexamethasone after IA-administration in the healthy carpal joint [8]. The very low value for *k*_*el*_ indicate that the half-life of dexamethasone in inflamed synovial fluid is similar to the half-life in plasma (the *t*_*1/2α*_ and *t*_*1/2β*_ in plasma was 0.6 h and 4.3 h, respectively). This is consistent with our preliminary calculations of dexamethasone half-life in inflamed synovial fluid (terminal half-life: 0.9–3.3 h) published elsewhere [31]

In both experimental and clinical studies joint swelling and lameness has been used as biomarkers after intra-articular glucocorticoid therapy in horses [3, 4, 6, 7]. LPS-challenge has been used previously to induce inflammation in the equine joint [32–36]. Doses up to 2.5 ng LPS per joint caused a mild to moderate local joint inflammation that resolved within 36 h, whereas doses above 125 ng also caused signs of systemic inflammation. This study demonstrates between animal lameness (HD_min_) variation after LPS challenge followed by saline injection (Fig. 5). This variation is not showed in the amplitude parameter, which is a consequence of the additional γ_1_-parameter and γ_2_-parameter. However, the γ-parameters were necessary to capture the peak and duration in lameness response. This was important to accurately fit the model to experimental data and estimation of model-parameters. Despite following strict procedures when injections were performed, incomplete intra-articular injection might have contributed to variation in response to the LPS-challenge and the dexamethasone treatment between the horses. Aspiration of synovial fluid followed by injection performed without resistance was used as confirmation of the needle placement within the joint cavity.

Lameness scores were lower after treatment with LPS + DSP than with LPS + saline. Five of the horses (Table 1) were treated with doses lower than the labelled dose 2–10 mg/joint [37]. In this study, the objective lameness assessment was a more sensitive method to evaluate lameness compared with the visual assessment. The inertial sensor based system has been validated for lameness detection and shown repeatability when used in a straight line as in this study and is comparable with optical based motion capture [20, 38, 39]. It has also been proposed that high intra- and interrater variability may bias and limit the usefulness of subjective lameness assessment [40–43]. The use of non-linear mixed effects model allowed fitting of a pharmacodynamic model to the sparse experimental objective lameness data and the quantitative information of the pharmacodynamics was estimated (Figs. 6 and 7, Table 3). Both the dexamethasone synovial fluid potency (*IC*_50_) and efficacy (*I*_*max*_) values were derived as well as model parameters for the LPS-challenge. The *IC*_50_ and *I*_*max*_ values are similar to what has been reported potency and efficacy values for glucose response in horses [44]. For all parameters for which the random component of the model was estimable, the BSV was relatively small (Tables 2 and 3). However, the shrinkage was high for the two pivotal pharmacodynamic parameters (*IC*_50_ and *I*_*max*_) indicating that the between subject variation for these two parameters was not estimable from our data set and it was impossible to estimate accurate individual *IC*_50_ and *I*_*max*_ (post hoc) values [24]. However we robustly estimated a typical value that should deserve attention.

Decreasing the dose may offer both benefits and disadvantages. Lower doses have lower risk for obvious systemic effects but also shorter duration of the local response in the joint. The use of lower doses may potentially increase the number of injections to uphold drug exposure over time. There are case reports describing septic arthritis following intra articular injections in the horse [45]. In the present study, totally 156 joint punctures were performed. There were no signs of septic arthritis during the study or in the following days. The result suggests that the risk for infection is low when aseptic injection techniques are used. This is also supported by a recent suggestion that infection is uncommon following joint injections [46]. However, septic arthritis is a severe condition and the risk should not be neglected. Higher doses would reduce the number of injections to maintain therapeutic concentrations in synovial fluid and consequently the risk for septic arthritis. However, there is some conflicting evidence for the risk of detrimental effects on articular cartilage at high glucocorticoid doses administered IA [47–49]. High doses also increase systemic exposure and might produce adverse systemic effects e.g. hyperglycaemia, hyperinsulinemia and potentially laminitis [50–52]. Further studies are warranted to evaluate if the benefit from lower doses outweigh the risks with repeated injections.

The variables JC (joint circumference) and ST (local skin temperature) were not statistically different between treatments. Both JC and ST have been used in response studies of anti-inflammatory drugs and there is conflicting evidence for decrease in those variables. Morphine has anti-inflammatory properties and decreased ST and JC in equine synovitis [33]. Despite that non-steroidal drugs might decrease ST, JC in horses or paw-swelling in dogs may not decrease which suggest that JC may not be a suitable biomarker for the anti-inflammatory responses in acute synovitis [53–56]. A likely explanation for the non-significant results in ST in this study is the low dexamethasone exposure following the lowest doses used combined with the relatively low power caused by study with only six horses. Consistent with this study significant lameness reduction and no decrease in JC after IA glucocorticoid administration has been reported elsewhere [4].

## Conclusion

The synovial disposition and the response to dexamethasone were characterised in an equine model of synovitis after a single IA administration of DSP. The inertial sensor based lameness scoring system was the most sensitive of the methods used to evaluate the response to dexamethasone in this study. The presented quantitative information can be used as input for both future research and programs intended to upheld integrity and horse welfare in horseracing and equestrian sports. Dexamethasone synovial fluid half-life was considered similar to that in plasma. A tentative potency and efficacy value for lameness reduction was proposed. Lameness was suppressed after treatment with dexamethasone despite doses lower than labelled doses (2–10 mg). Low doses of DSP combined with short half-life of dexamethasone in synovial fluid following IA administration will result in short duration of response, which must be considered in clinical treatment.

## Data Availability

The datasets used and/or analysed during the current study are available from the corresponding author on reasonable request.
